# Molecular characterisation of emerging tacheng tick virus 2 in ticks collected from livestock and dogs in Türkiye

**DOI:** 10.1007/s10493-026-01149-4

**Published:** 2026-06-11

**Authors:** Ender Dincer, Mehmet Ozkan Timurkan, Sebahattin Akca, Fatma Nur Yuce

**Affiliations:** 1 Faculty of Veterinary Medicine, Department of Virology, Dokuz Eylül University, Izmir, Türkiye; 2 Faculty of Veterinary Medicine, Department of Virology, Atatürk University, Erzurum, Türkiye; 3 Erzurum Veterinary Control Institute, Department of Virology, Ministry of Agriculture and Forestry, Erzurum, Türkiye

**Keywords:** Phleboviruses, Tacheng tick virus 2, Tick-borne diseases, Zoonoses, Türkiye

## Abstract

The aim of this study was to investigate the presence of TcTV-2 in ticks collected from ecologically diverse regions of Türkiye. Between 2023 and 2024, ticks were collected from sheep, goats, cattle, and dogs across 14 provinces representing different ecological zones. A total of 1051 ticks have been collected and sorted into 10 different species. The most common ones were *Haemaphysalis parva* (26.4%), *Rhipicephalus turanicus* (23.7%), *Dermacentor marginatus* (11.6%), and *Rhipicephalus sanguineus* (8.1%), among others. The TcTV-2 RNA was detected in 15 (7.5%) of 199 tick pools using nRT-PCR. The virus was identified in pools consisting of the following species: *Haemaphysalis punctata*, *Dermacentor marginatus*, *Rhipicephalus sanguineus*,* Haemaphysalis parva*, *Hyalomma marginatum*, *Rhipicephalus bursa*, and *Hyalomma anatolicum excavatum*. The polyphyletic distribution of the Turkish isolates suggests that these strains do not originate from a single source; rather, they likely reflect multiple introduction events or the accumulation of genetic diversity through prolonged local circulation. In particular, the observation that some isolates are clearly separated from other Turkish sequences further supports the heterogeneous structure of the viral population within Türkiye. Our findings suggest that TcTV-2 may occur in a wider range of tick species and ecological regions in Türkiye; however, further temporal and evolutionary analyses are required to assess potential expansion dynamics.

## Introduction

Ticks (class Arachnida, subclass Acari) are blood-feeding ectoparasites of vertebrates. So far, scientists have found about 900 different types of ticks around the world. These ticks are usually divided into two main groups: hard ticks (family Ixodidae) and soft ticks (family Argasidae) (Estrada-Pena and Fuente 2014). Ticks are known as the second most significant arthropod group after mosquitoes in terms of their role in transmitting a wide range of pathogens, including parasites, bacteria, and viruses. Despite various control strategies, global warming and anthropogenic activities, particularly deforestation and converting wildlife habitats into agricultural land, have facilitated the expansion of tick populations into new geographic areas. Consequently, the increasing frequency of human–tick interactions poses growing public health concerns (Socha et al. 2022).

The families Phenuiviridae and Flaviviridae have a variety of tick-borne RNA viruses with zoonotic potential, such as Kyasanur Forest disease virus, Alkhumra virus, and Crimean-Congo haemorrhagic fever virus (CCHFV) (Charnel et al. 2004; Kapuscinski et al. 2021). In recent decades, the new genetic and molecular techniques, including high-throughput and deep sequencing, have led to the identification of novel tick-borne viral species and strains. New emerging and re-emerging viruses, such as Tacheng tick virus-1 (TcTV-1), Tacheng tick virus 2 (TcTV-2), Tamdy virus (TAMV), Songling virus, Yezo virus, and Jingmen tick virus (JMTV), have been reported in numerous tick species from multiple countries, including China, Türkiye, Kenya, Poland, Japan, Russia, and Georgia (Liu et al. 2020; Dong et al. 2021; Moming et al. 2021; Ji et al. 2023; Zhang et al. 2025; Qin et al. 2014; Matsumura et al. 2024; Dincer et al. 2023; Ogola et al. 2022; Tupota et al. 2023).

TcTV-2 (family Phenuiviridae, genus Uukuvirus) is a novel emerging tick-borne RNA virus that has a genome with three segments, namely L (large), M (medium), and S (small), first identified in RNA sequencing studies of *Dermacentor marginatus* ticks in China (Li et al. 2015). Subsequently, in 2019, TcTV-2 was isolated from a tick-bitten patient showing symptoms of severe fatigue, fever, anorexia, and vomiting in China. TcTV-2 was also detected in various tick species, including Dermacentor silvarum, Dermacentor nutalli, *Dermacentor marginatus*, and *Hyalomma asiaticum* collected from the patient’s living environment (Dong et al. 2021). Furthermore, to date, the distribution of TcTV-2 has been reported in a broad geographical area, including China, Kazakhstan, Eastern Europe, and Türkiye, and in tick species such as *D. marginatus* (Li et al. 2025), *Hy. marginatum (*Brinkmann et al. 2018), *Hy. scupense*, *D. reticulatus* (Bratuleanu et al. 2022), and *Hy. asiaticum* egg batches and blood samples of wild animals such as badgers and red foxes. Importantly, Chinese herdsmen’s serum samples also contained TcTV-2 (Jia et al. 2024). These results indicate that TcTV-2 has the potential to infect humans. However, the pattern of TcTV-2 infection is poorly defined. Therefore, laboratory studies and epidemiological surveillance are essential to elucidate the biology of TcTV-2 in vector ticks and susceptible hosts, including humans, livestock, and wildlife.

Türkiye is situated at the crossroads of three continents, namely Europe, Asia, and Africa, and lies along major migratory bird flyways. These birds can transfer various ticks across continents, facilitating the transboundary spread of tick-borne viruses. The ecological factors, such as climate and vegetation, contribute to the high diversity of tick vectors and their animal hosts. CCHFV is the most well-known in Türkiye, with a reported 1,318 human cases between 2008 and 2017 (Ergünay et al. 2020a, b). In recent decades, novel emerging tick-borne viruses, including JMTV, TcTV-1, TAMV, and TcTV-2, have been found and genetically characterised in ticks investigated in both domestic and wild animals between 2019 and 2023 (Dincer et al. 2019; 2022; 2023; Ergünay et al. 2020a, b). However, geographical distribution, tick vectors, and potential wild and domestic animal reservoirs were largely undetected, with their potential public health impact in Türkiye. In this study, we conducted a comprehensive tick surveillance effort to detect and characterise TcTV-2 in ticks collected from ecologically and geographically diverse regions across Türkiye.

## Materials and methods

### Sample collection and processing

**Fig. 1:**
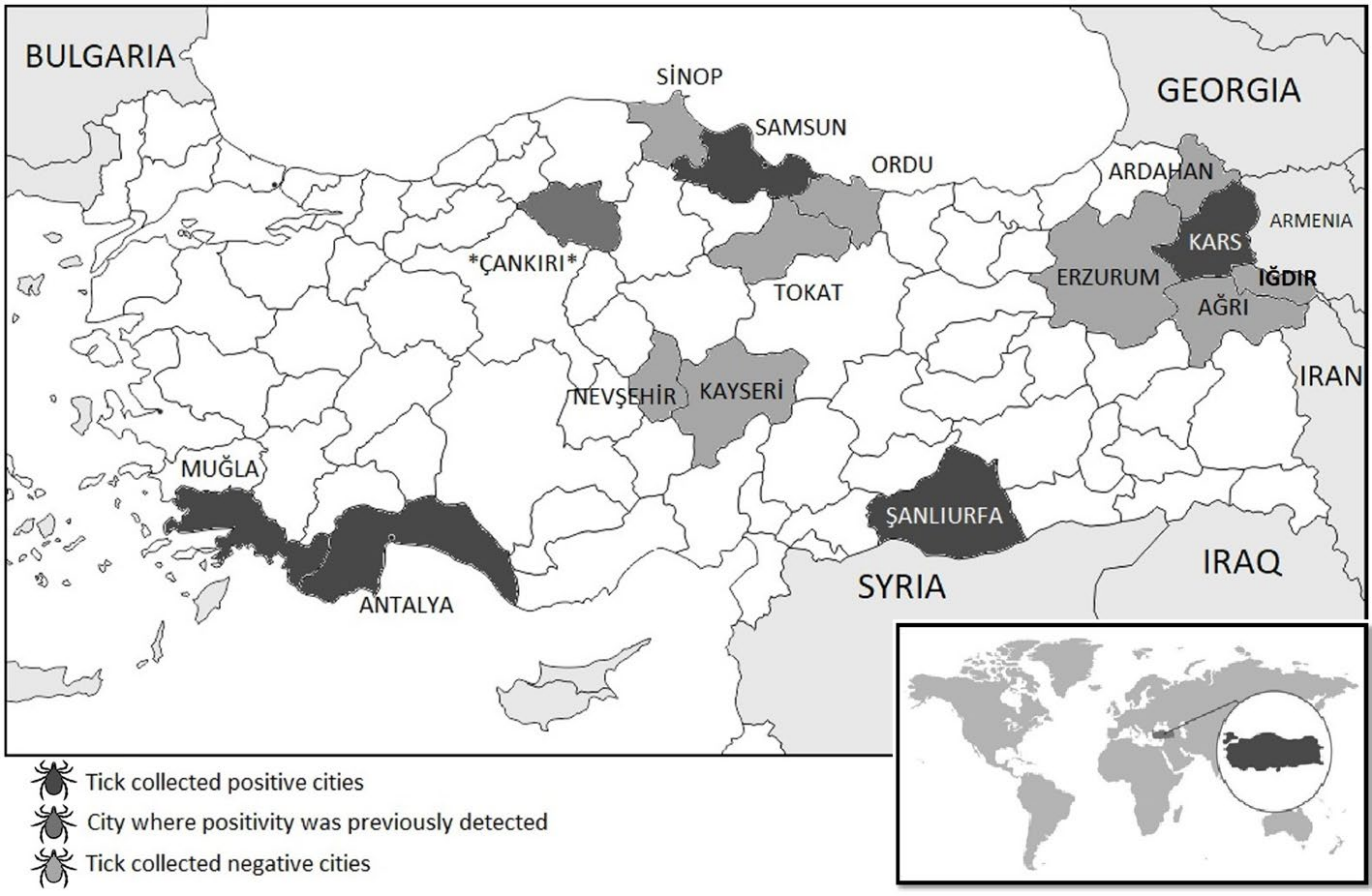
The illustrative map demonstrates distributions of TcTV-2 documented in Türkiye and the geographical locations of tick collection sites. (⁎ Çankırı province where TcTV − 2 was detected in 2022)

A total of 1051 ticks were collected from sheep, goats, cattle, and dogs visiting pet clinics across Türkiye. The tick specimens investigated in the present study consisted of field-collected host-seeking or questing ticks, those taken from domestic animals, and the cooperation of the caretakers. The six locations of Türkiye were selected as study areas: Kars, Ağrı, Iğdır, Erzurum, and Ardahan in Eastern Anatolia; Sinop, Samsun, Tokat, and Ordu in the Black Sea region; Şanlıurfa in Southeastern Anatolia, Kayseri and Nevşehir in Central Anatolia; Muğla in the Aegean region, and Antalya in the Mediterranean region, between 2022 and 2023 (Fig. 1). Tick species were identified under a stereomicroscope using taxonomic keys (Estrada-Pena et al. 2004), and the ticks were subsequently grouped into pools of 1 to 10 based on their size and blood-feeding status. The pools were homogenised using vortexing with 4.5- or 7.0-mm tungsten carbide beads (QIAGEN, Hilden, Germany) with 200–500 µL of RPMI medium, and then samples were placed in a homogeniser. The homogenised tick tissue samples were then stored at -80 °C until nucleic acid extraction.

**Fig. 2:**
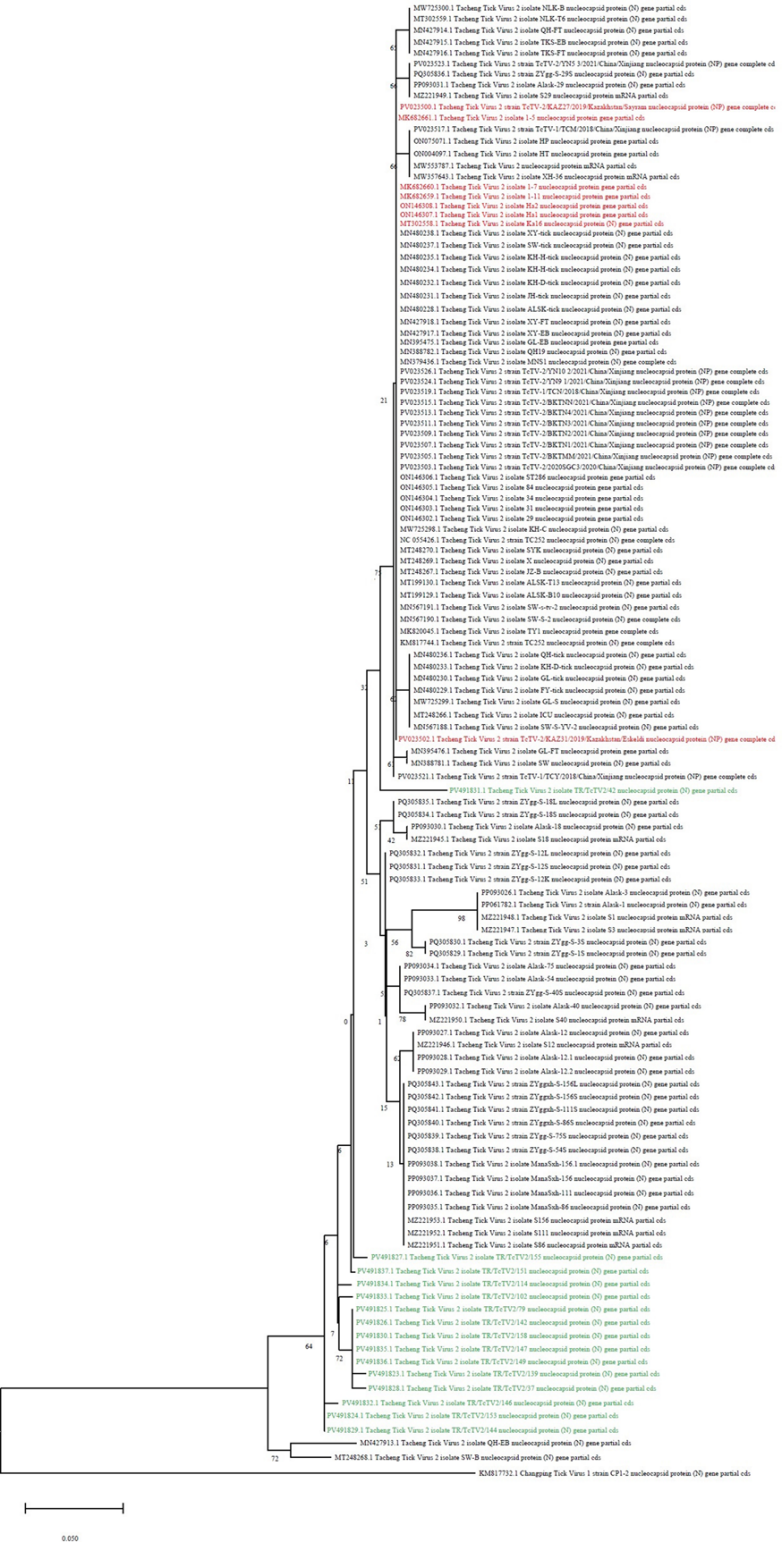
Phylogenetic analysis of TcTV-2 partial S segment sequences (227 nucleotides) was conducted using the maximum likelihood method. The tree is constructed using the Tamura 3-parameter model for 1000 replications. Viruses are indicated by GenBank accession number, abbreviation, isolate/strain identifier, host/tick species, and country of origin. In this analysis, Kazakh sequences are shown in red, Chinese sequences in black, and Turkish sequences in green. The Changping tick virus is used as an outgroup: TcTV-2, the Tacheng tick virus 2

### RNA extraction and virus detection

 After each grinding solution was centrifuged at 6 000 rpm for 10 min, 200 µL of supernatant was placed in a separate, clean 1.5 mL centrifuge tube. According to the manufacturer’s instructions, the nucleic acid extraction was performed using the High Pure Viral Nucleic Acid Kit (Roche Diagnostics, Germany). The reverse transcription step was performed using the RevertAid First Strand cDNA Synthesis Kit (Thermo Fisher Scientific, Germany) according to the manufacturer’s instructions. To prevent cross-contamination, negative extraction controls were established in every 15 samples. Subsequently, nRT-PCR was performed using primer sets (Out-F1: 5’–ATCTCCTCAACGGCAACTAT-3’, Out-R1: 5’GACATGCGGTTCTTCATTTT-3’, In-F2: 5’-TCAACGGCAACTATGAGGAT-3’, and In-R2: 5’-CTGGCTTGTATTGGAAGGAC-3’) targeting segment S of TcTV-2, as stated previously (6). The first and second steps of PCRs were set up as a final volume of 30 µL with a Taq DNA polymerase kit (Thermo Fisher Scientific, Germany) according to the manufacturer’s instructions. The PCRs were carried out at 95 °C for 4 min, followed by 40 cycles of 94 °C for 45 s, 50 °C for 1 min, and 72 °C for 1 min, and a final extension of 72 °C for 5 min. The obtained PCR products (252 base pairs in length) were separated using 1% agarose gel with SYBR Safe DNA gel stain (Thermo Fisher Scientific, Germany) and visualised by an imaging system (a ChemiDoc XRS+ imaging system (Bio-Rad, Germany)). We used DNase/RNase-free water for each PCR to prevent contamination. In addition, viral nucleic acid extraction and nRT-PCR steps were carried out using biosafety level 2 class cabinets.

### Sequencing and data analyses

The sequence analysis of PCR products produced by a commercial kit (GeneJET; Thermo Scientific, USA) was performed in an ABI PRISM 3500xL Dx Genetic Analyser (Thermo Fisher Scientific, USA) using the second-step PCR primers. The raw sequences’ sequence alignment and identity analysis were investigated using BioEdit (version 7.7.1) bioinformatics software (Hall 1999). We employed the maximum-likelihood tree in the Tamura-3-parameter model in MEGA X software 10.2.6 to elucidate nucleotide comparisons. We constructed the phylogenetic tree using bootstrap analysis (1000 replicates) (Kumar et al. 2018).

## Results

### Tick collection and identification of TcTV-2

A total of 1051 ticks, comprising 10 species, were collected from sheep, goats, cattle, and dogs in Türkiye (Table 1). The examination showed that the ticks included *Haemaphysalis parva* (*n* = 278; 26.4%), *Rhipicephalus turanicus* (*n* = 250; 23.7%), *Dermacentor marginatus* (*n* = 122; 11.6%), *Haemaphysalis punctata* (*n* = 107; 10.1%), and other species. A total of 199 tick pools consisted of southeastern Anatolian (14; 7.3%), eastern Anatolian (67; 63%), central Anatolian (15; 7.5%), Black Sea region (69; 34.5%), Aegean (6; 3%), and Mediterranean (69; 34.5%; 8%) provinces (Table 1).

A total of 15 tick pools (15/199, 7.5%) were found positive for TcTV-2 with nRT-PCR targeting the S segment. Provinces with positive tick pools occurred in Muğla (n=1, 6.6%); Kars (n=2, 13.3%); Antalya (n=8, 53.3%); Samsun (n=2, 13.3%); and Şanlıurfa (n=1, 6.6%), respectively. *R. bursa* ticks collected from goats and sheep in Antalya province (Mediterranean region) showed the highest positivity rate. We detected TcTV-2 in the female and male pools of *D. marginatus* collected from Samsun and Kars provinces. Moreover, the single male *D. marginatus* specimen obtained from Antalya province was positive for TcTV-2 (Table 2). The phylogenetic tree (Fig. 2) did not show a clear geographical clustering among the isolates. Sequences originating from Türkiye were not grouped within a single clade but were instead distributed throughout the phylogenetic tree. In particular, some isolates (e.g., PV491831.1) were located far from other sequences from Türkiye. Similarly, isolates from Kazakhstan and China did not form monophyletic groups but were instead distributed along different branches. These findings indicate the absence of a distinct phylogenetic structure associated with geographic origin among the analysed sequences. Genetic distance analysis demonstrated low overall variability among TcTV-2 isolates. The average genetic divergence across all isolates was approximately 1.8%, indicating limited genetic diversity. However, two Chinese isolates (MN427913 and MT248268) formed a small subcluster, showing approximately 6–7% divergence from the main group. In contrast, the genetic distance between the main TcTV-2 cluster and the outgroup (Changping tick virus) was approximately 37%. These results support that all analysed isolates belong to a single genetic lineage with no clear evidence of geographically distinct sublineages. Furthermore, the phylogenetic analysis was based on a short fragment of the nucleocapsid (N) gene (~227 bp), which represents an important constraint on phylogenetic resolution.

**Table 1:** Distribution of the tick samples according to species and collection areas

	Southeastern Anatolia		Eastern Anatolia										Central Anatolia				Black sea region								Aegean		Mediterranean		
	Şanlıurfa		Ardahan		Kars		Ağrı		Erzurum		Iğdır		Kayseri		Nevşehir		Sinop		Tokat		Ordu		Samsun		Muğla		Antalya		
Species	♀	♂	♀	♂	♀	♂	♀	♂	♀	♂	♀	♂	♀	♂	♀	♂	♀	♂	♀	♂	♀	♂	♀	♂	♀	♂	♀	♂	
																													Total
Hyolamma marginatum	0	0	0	0	0	0	0	0	0	0	0	0	0	0	0	3	0	0	8	0	0	0	0	0	14	19	0	0	44(4.1%)
Haemaphysalis parva	0	0	21	17	34	48	27	64	2	0	18	47	0	0	0	0	0	0	0	0	0	0	0	0	0	0	0	0	278(26.4%)
Rhipicephalus sanguines	40	46	0	0	0	0	0	0	0	0	0	0	0	0	0	0	0	0	0	0	0	0	0	0	0	0	0	0	86(8.1%)
Dermacentor marginatus	0	0	0	0	10	4	0	0	26	44	2	7	0	0	0	0	0	0	0	0	0	0	12	16	0	0	0	1	122(11.6%)
Rhipicephalus turanicus	0	0	0	0	0	0	0	0	0	0	0	0	34	20	0	0	123	73	0	0	0	0	0	0	0	0	0	0	250(23.7%)
Haemaphysalis punctata	0	0	0	0	0	0	0	0	0	0	0	0	0	0	0	0	0	0	0	0	0	0	84	23	0	0	0	0	107(10.1%)
Hyolamma analiticum excavatum	0	0	0	0	0	0	0	0	0	0	0	0	17	19	0	4	0	0	0	0	0	0	0	0	0	0	1	0	41(3.9%)
Ixodes ricinus	0	0	0	0	0	0	0	0	0	0	0	0	0	0	0	0	0	0	21	4	12	2	3	0	0	0	0	0	42(3.9%)
Hyolamma analiticum	4	0	0	0	0	0	0	0	0	0	0	0	0	0	0	0	0	0	0	0	0	0	0	0	0	0	0	0	4(0.3%)
Rhipicephalus bursa	0	0	0	0	0	0	0	0	0	0	0	0	0	0	0	0	0	0	0	0	0	0	0	0	0	0	28	49	77(7.3%)
Total	44	46	21	17	44	52	27	64	28	44	20	54	51	39	0	4	123	73	29	4	12	2	99	39	14	19	29	50	1.051
	90(8.5%)		38(3.6%)		96(9.1%)		91(8.6%)		72(6.8%)		74(7.4%)		90(8.5%)		4(0.3%)		196(19.6%)		33(3.1%)		14(1.3%)		138(13.1%)		33(3.1%)		79(7.5%)		
Pool	14(7.3%)		8(4%)		24(12%)		12(6%)		12(6%)		11(5.5%)		14(7%)		1(0.5%)		30(15%)		7(3.5%)		6(3%)		26(13%)		6(3%)		16(8%)		199

**Table 2:** Characteristics of TcTV–2–positive tick pools

Code	Location	Species	Pool Size	Host	GeneBank Accession No
37	Samsun	*Haemaphysalis* * punctata*	8♀	Sheep	PV491828
114		*Dermocentor marginatus*	9♂		PV491834
42	Kars	*Dermocentor marginatus*	4♀		PV491831
102		*Haemaphysalis* * parva*	12♂		PV491833
79	Muğla	*Hyalomma marginatum*	4♀	Cattle	PV491825
139	Şanlıurfa	*Rhipicephalus sanguines*	10♂	Dog	PV491823
142	Antalya	*Rhipicephalus bursa*	4♀	Sheep	PV491826
144			4♂		PV491829
147			2♀	Goat	PV491835
149			5♂		PV491836
153			5♂	Sheep	PV491824
155			3♀		PV491827
158			4♀	Goat	PV491830
146		*Hyalomma analiticum excavatum*	1♀	Goat	PV491832
151		*Dermacentor marginatus*	1♂	Sheep	PV491837

†♀: female; ♂: male

## Discussion

In this survey, we investigated the presence of TcTV-2 in ticks collected from livestock (sheep, goats, and cattle) and dogs in Türkiye. A total of 1, 051 ticks were collected from livestock and dogs from different ecogeographical areas, including southeastern Anatolia (Şanlıurfa province), the Aegean region (Muğla province), eastern Anatolia (Kars, Igdır, Ardahan, and Erzurum provinces), the Black Sea region (Sinop, Tokat, and Samsun provinces), and central Anatolia (Kayseri and Nevşehir provinces) in 2023 and 2024. 199 tick pools were screened for TcTV-2 using previously reported nRT-PCR (Dong et al. 2021), and 15 (7.5%) tick pools were identified as positive for TcTV-2.

In the past decade, emerging and re-emerging viruses, particularly arthropod-borne viruses, have expanded their geographical range. The *Phenuiviridae* family has recently been augmented with new members, including two tick-borne phleboviruses: Severe Fever with Thrombocytopenia Syndrome Virus (SFTSV) and Heartland Virus. Both viruses have been reported in patients with multiple organ damage in China and the USA (Yu et al. 2011; McMullan et al. 2012). TcTV-2, another new member of the family *Phenuiviridae*, was initially reported in *D. marginatus* ticks in China (Qin et al. 2014). In 2019, it was also isolated from a patient with a history of tick bites presenting clinical symptoms of malaise, rash, and vomiting in the Xinjiang Uygur Autonomous Region of China. Further studies showed that TcTV-2 has been circulated in various tick species, including *D. silvarum*,* D. marginatus*,* Hy. asiaticum*, and *D. nutalli* in the same region. These findings suggest that TcTV-2 is an emerging tick-borne virus that has raised public health concerns. Subsequent detection of TcTV-2 occurred in *D. marginatus* and *Hy. asiaticum* ticks in Kazakhstan (Jia et al. 2022). The first report of TcTV-2 in Europe was described in *D. reticulatus* ticks using metagenome-based nanopore analysis (Ergunay et al. 2023). In 2023, more studies showed that TcTV-2 was present in different body fluids of patients bitten by ticks, as well as in blood and spleen samples from wild animals like red foxes and badgers and in egg batches of *Hy. asiaticum* in China. Additionally, researchers evaluated seroconversion in serum samples from herders (Jia et al. 2024).

The TcTV-2 has been initially documented *in Hy. marginatum* ticks, the main vector of CCHFV, by a metagenomic survey conducted in the Aegean region (Muğla province) in Türkiye (Brinkmann et al. 2018). Later, TcTV-2 was discovered in both male and female *D. marginatus* ticks taken from cattle using nRT-PCR that focused on the S segment in central Anatolia (Çankırı Province) in 2022. The present study revealed that TcTV-2 has a wider circulation region than previously thought in Türkiye. Additionally, finding TcTV-2 in both female and male ticks, as shown in earlier studies, indicates that the virus can be passed from parent ticks to their eggs and also between different life stages of the ticks. The Antalya and Şanlıurfa provinces, where TcTV-2 was detected, are majorly visited locations for local and international tourists. These areas have various ecological zones, including natural lakes, forests, wild bird resting areas and mountains. Especially, Antalya is an important destination mainly for ecotourism, hiking routes, camping sites, and natural life parks (Manavoglu and Yıldırım, 2020). Engaging in these outdoor activities may lead humans to encounter various tick species (Koc et al. 2015), which could increase the risk of exposure to tick-borne viruses (Dincer et al. 2022). In particular, humans and ticks may coincide during the warmer months. The diverse ecosystems of Şanlıurfa, ranging from semi-arid areas to fertile plains, contribute to the presence of a wide variety of tick species. Moreover, the agricultural activities and the movement of livestock across regions further facilitate tick-human interactions (Bakırcı et al. 2019). Considering these factors, obtaining TcTV-2 in ticks from this region is of particular concern, as it may indicate local viral circulation (Dincer et al. 2017; 2019; 2022), which could be amplified by increased human and animal movement across borders. In this study, we showed that 8 out of 16 tick pools tested positive for TcTV-2, with the highest positivity rate observed in Antalya compared to other provinces. The repeated detection of TcTV-2 in *Hy. marginatum* tick pools in Muğla province also suggests that viral circulation may be ongoing in this region, prompting further investigation into the dynamics of tick-borne viruses and their potential vectors (Brinkmann et al. 2018; Dinçer et al. 2017). Another province reported TcTV-2 in ticks; Kars is located on the border of Georgia and Armenia. More studies, especially those looking at the entire genetic makeup of the tick population, have found TcTV-2 in ticks like *D. marginatus*,* D. reticulatus*, and *H. punctata* in Georgia, Poland, and Ukraine. These results further support the notion that the presence of TcTV-2 in the Kars and Samsun provinces of Türkiye is expected. Both provinces are located in the same ecogeographical zone as Eastern Europe and the Black Sea region, where tick species commonly inhabit and circulate. As a result, the potential for viral transmission through ticks in these regions is high, given the shared environmental and ecological conditions that facilitate the presence of such tick species (Ergünay et al. 2023; 2024).

The phylogenetic data presented in Fig. 2 did not reveal a clear phylogeographic structure among the TcTV-2 isolates. The absence of distinct and well-supported lineages associated with sequences from Türkiye, Kazakhstan, and China may suggest that the currently available sequences do not exhibit strong geographic clustering. The distribution of Turkish isolates across different branches may indicate genetic heterogeneity among circulating strains; however, interpretations regarding multiple introduction events or long-term local circulation should be made cautiously due to the limited phylogenetic resolution of the dataset. Likewise, the scattered placement of sequences from Kazakhstan and China could reflect a broad ecological distribution of the virus, although definitive conclusions regarding interregional movement or gene flow cannot be drawn from the present data. Potential ecological factors, including tick dispersal, host animal movement, migratory birds, and trade, may contribute to virus dissemination, but these hypotheses require further investigation. In addition, the relatively low bootstrap support values across several branches and the use of a short genomic region may limit the robustness of the phylogenetic inferences. Overall, the findings suggest genetic diversity among TcTV-2 isolates without clear clustering according to geographic origin. Future studies involving larger sample sizes and whole-genome analyses will be important for improving our understanding of the evolutionary dynamics and geographic distribution of this virus.

This study has some limitations and concerns regarding the interpretation of the findings. Due to the study design and the pooling and analysis of tick samples, the exact source of the detected sequences could not be definitively determined, and the possibility of an environmental origin cannot be ruled out. The detection of TcTV-2 sequences in ticks collected from cattle, dogs, goats, and sheep (Table 2) suggests that, in some or all cases, parasitised vertebrates may be a possible source. This necessitates screening vertebrates to identify potential exposure and viraemic animal hosts. These findings should be considered indicators of virus presence and exposure. In this context, virus isolation, demonstration of viral replication in ticks, investigation of transstadial and transovarial transmission, and experimental transmission studies are of great importance.

## Conclusion

TcTV-2 was a recently reported emerging tick-borne RNA virus in ticks, wild animals, and humans in the different geographical areas. However, our understanding of tick vectors and their role in wildlife, domestic animals, and migratory birds remains inadequate. In the present study, we suggested the presence of TcTV-2 in multiple tick species collected from ecologically diverse regions of Türkiye. Importantly, ticks, including *H. parva*, *H. punctata*, *Hy. a. excavatum*, *R. bursa*, and *R. sanguineus*, were firstly noted for TcTV-2 in Türkiye. Our data confirm that TcTV-2 has a broader geographic and vector distribution than previously reported. Comprehensive surveillance is needed to elucidate its global distribution and to prevent potential local or large-scale outbreaks, particularly in regions where the virus was previously detected.

## Data Availability

The sequence data obtained from this study are freely available within the NCBI GenBank database under accession numbers PV491823 - PV491837. The datasets generated during the current study are available in the Results section.
