# Publisher Correction: Molecular characterisation of emerging tacheng tick virus 2 in ticks collected from livestock and dogs in Türkiye

**DOI:** 10.1007/s10493-026-01156-5

**Published:** 2026-07-21

**Authors:** Ender Dincer, Mehmet Ozkan Timurkan, Sebahattin Akca, Fatma Nur Yuce

**Affiliations:** 1 Faculty of Veterinary Medicine, Department of Virology, Dokuz Eylül University, Izmir, Türkiye; 2 Faculty of Veterinary Medicine, Department of Virology, Atatürk University, Erzurum, Türkiye; 3 Erzurum Veterinary Control Institute, Department of Virology, Ministry of Agriculture and Forestry, Erzurum, Türkiye

**Publisher Correction: Experimental and Applied Acarology (2026) 97:6**

10.1007/s10493-026-01149-4

In this article, the paragraphs that belong to the "Materials and methods" section were incorrectly displayed in the section "Conclusions".

The original article has been corrected.

